# Physically informed Monte Carlo simulation of dual-wedge prism-based spectroscopic single-molecule localization microscopy

**DOI:** 10.1117/1.JBO.29.S1.S11502

**Published:** 2023-10-03

**Authors:** Wei-Hong Yeo, Cheng Sun, Hao F. Zhang

**Affiliations:** a Northwestern University, Department of Biomedical Engineering, Evanston, Illinois, United States; b Northwestern University, Department of Mechanical Engineering, Evanston, Illinois, United States

**Keywords:** Monte Carlo simulation, spectroscopy, super-resolution microscopy, single-molecule localization microscopy

## Abstract

**Significance:**

The dual-wedge prism (DWP)-based spectroscopic single-molecule localization microscopy (sSMLM) system offers improved localization precision and adjustable spectral or localization performance, but its nonlinear spectral dispersion presents a challenge. A systematic method can help understand the challenges and thereafter optimize the DWP system’s performance by customizing the system parameters to maximize the spectral or localization performance for various molecular labels.

**Aim:**

We developed a Monte Carlo (MC)-based model that predicts the imaging output of the DWP-based sSMLM system given different system parameters.

**Approach:**

We assessed our MC model’s localization and spectral precisions by comparing our simulation against theoretical equations and fluorescent microspheres. Furthermore, we simulated the DWP-based system using beamsplitters (BSs) with a reflectance (R):transmittance (T) of R50:T50 and R30:T70 and their tradeoffs.

**Results:**

Our MC simulation showed average deviations of 2.5 and 2.1 nm for localization and spectral precisions against theoretical equations and 2.3 and 1.0 nm against fluorescent microspheres. An R30:T70 BS improved the spectral precision by 8% but worsened the localization precision by 35% on average compared with an R50:T50 BS.

**Conclusions:**

The MC model accurately predicted the localization precision, spectral precision, spectral peaks, and spectral widths of fluorescent microspheres, as validated by experimental data. Our work enhances the theoretical understanding of DWP-based sSMLM for multiplexed imaging, enabling performance optimization.

## 1 Introduction

Single-molecule localization microscopy (SMLM) allows for sub-diffraction-limit imaging of biological structures down to 10 nm,^1^^–^^3^ with spectroscopic SMLM (sSMLM) extending this capability to imaging multiple molecular contrasts through analysis of single molecular fluorescence emission spectra.^4^^–^^10^ In sSMLM, a dispersive component, such as a grating^4^^–^^7^ or a prism,^8^^–^^10^ divides photons from each blinking event to form a spatial image for localization and a spectral image for spectroscopic analysis. Hence, sSMLM can identify each fluorophore based on its characteristic spectrum with nanoscopic spatial precision.

The main constraint limiting higher performance in both SMLM and sSMLM is the photon budget.^11^^,^^12^ Gratings used in sSMLM are associated with high transmission losses (∼30%), reducing the photon budget, which worsens localization and spectral precisions.^11^^–^^13^ By contrast, prism-based sSMLM has lower transmission losses but a higher aberration, which causes imaging artifacts.^13^ To address these issues, we recently developed a dual-wedge prism (DWP)-based sSMLM that features lower transmission losses, reduced aberrations, the ability to perform highly multiplexed imaging within an extended spectral range, and an adjustable splitting ratio for spatial and spectral imaging.^13^ Using DWP-based sSMLM poses challenges, such as a non-uniform spectral dispersion, which is poorly understood, but also benefits, such as an adjustable beamsplitter (BS) ratio that allows us to maximize the localization or spectroscopic performance.

First, the DWP module provides a higher transmission efficiency than the grating, allowing us to expand our highly multiplexed imaging capabilities through increased localization and spectral precision. Although the DWP module exhibits a nonlinear spectral dispersion, we previously assumed a constant spectral precision in our theoretical analysis of DWP-based sSMLM because we restricted its operation to a limited spectral range of 650 to 800 nm.^12^^,^^13^ When we extend the spectral range to 450 to 800 nm, which is possible with the DWP due to its higher transmission efficiency throughout the entire spectral range, the spectral dispersion may no longer be considered constant because spectral precision is a function of spectral dispersion.^12^

Next, the splitting ratio of the BS within the DWP module may be tailored for specific imaging requirements for imaging fluorophores with different emission spectral bandwidths. This is because adjusting the splitting ratio in the DWP module leads to a varying tradeoff between spatial and spectral precisions because the total photon budget is shared between the spatial and spectral channels. Although several simulation packages have been developed to help users optimize SMLM imaging conditions and understand SMLM imaging results,^14^^–^^20^ none of the packages directly simulate the spectra of the fluorophores. In addition, sSMLM imaging parameters, such as the choice of fluorophores and splitting ratios, affect the photon budget, which in turn affects the imaging performance quantified by the localization and spectral precisions. The relationship between the imaging conditions and localization and spectral precisions is intrinsically connected but poorly understood in DWP-based sSMLM.

Therefore, there is a need to thoroughly investigate the tradeoff between the extended spectral range and the additional flexibility of variable beamsplitting ratios afforded by the DWP module. In this work, we develop a Monte Carlo (MC) simulation of the 2D-DWP imaging process of individual fluorophore blinking events and determine each fluorophore’s spectral and spatial precisions. Our MC simulation of the 2D-DWP imaging process provides valuable insights into optimizing experimental conditions for imaging, eliminating the need for trial and error. By accurately estimating the localization precision and spectral precision with different splitting ratios, we can directly determine the ideal experimental parameters with different experiments. This optimization enables the use of more fluorophores in a single experiment, unlocking the potential to analyze intricate biological interactions at the nanoscale. The implementation of these refined experimental conditions for multiplexed experiments will enhance our ability to study and understand more complex biological interactions.

## 2 Methods

### 2.1 Image Formation Process

**Fig. 1:**
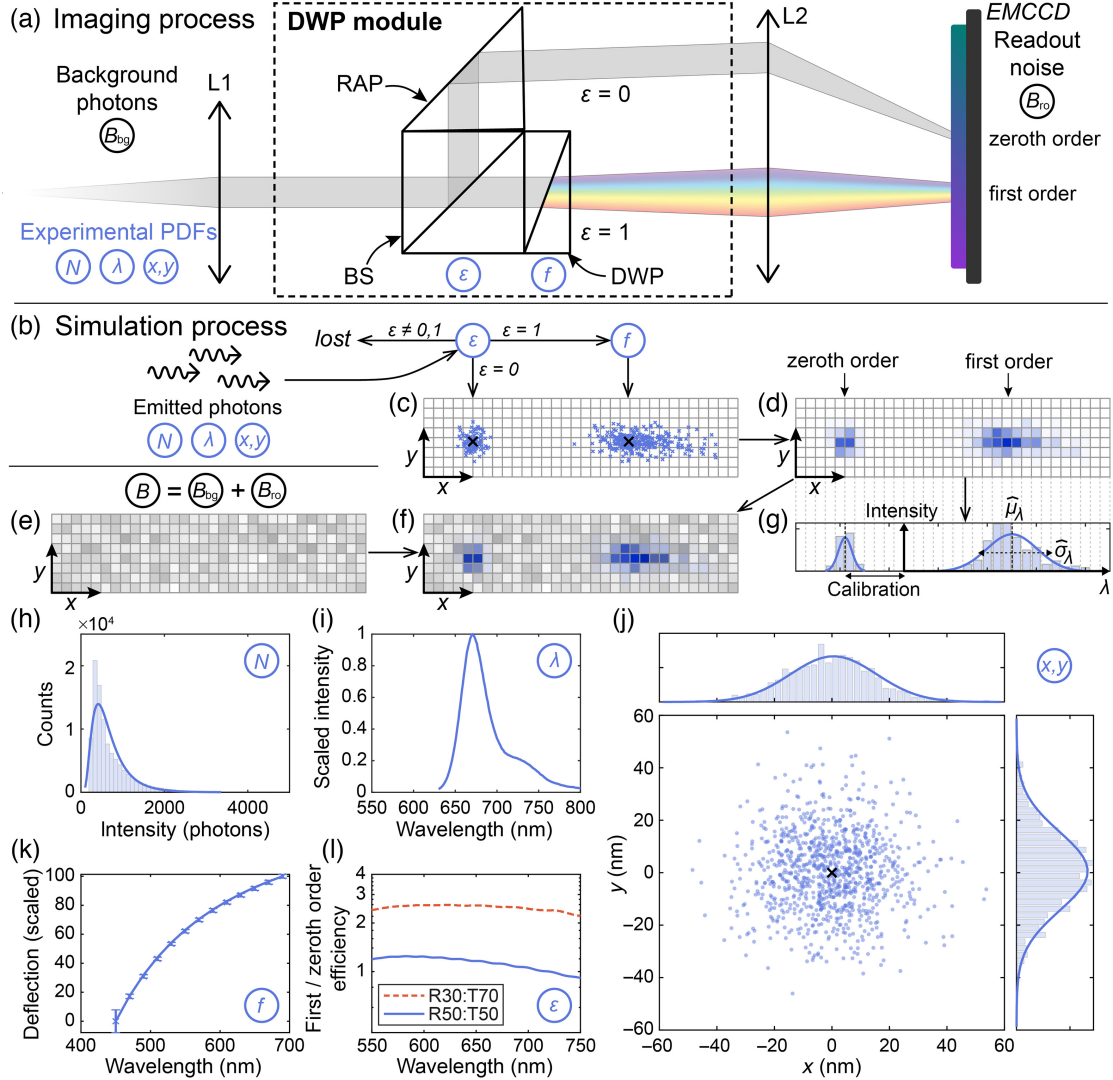
Illustration of the 2D-DWP image formation and MC simulation steps. (a) Schematic of the 2D-DWP image formation; (b) photon emission simulation process; (c) a simulated image of the photon distribution on the EMCCD following a Gaussian distribution of photon wavelengths; (d) corresponding EMCCD-detected image for photon distribution shown in panel (c); (e) simulated EMCCD background, including background and readout noises; (f) combined image from panels (d) and (e); (g) 1D fitting the spectral peak and with from spectral profile, where the zeroth order image provides the reference needed to obtain the corresponding first order spectral information; (h) measured photon count distribution of AF647 with a 10 ms exposure time; (i) measured bulk emission spectrum of AF647; (j) measured x and y variations of the individual photons due to the diffraction limit, with x- and y-summed histograms shown; (k) measured spectral deflection characteristics of the DWP module, and (l) measured ratios of first order/zeroth order efficiency in the DWP module when different BSs are used. RAP, right-angle prism; BS, beamsplitter.

The MC simulation modeled our current 2D-DWP-based sSMLM system as previously reported.^13^ We used two excitation lasers (532 nm, Exlsrone-532-200, Spectra-Physics; and 647 nm, 2RU-VFL-P-2000-647-B1R, MPB Communications) and an inverted microscope body (Ti2-E, Nikon) equipped with a 100× total-internal reflection fluorescence (TIRF) objective (CFI Apochromat TIRF 100XC Oil, Nikon). Figure 1(a) shows the photon flow in the detection path. The emitted photons from the fluorescent sample pass through a DWP module, consisting of a BS prism (BS010 for R50:T50; and BS049 for R30:T70, Thorlabs), a right-angle prism (RAP) (84-514, Edmund Optics), and a custom-made DWP.^13^ The BS divides the photons into a spatial imaging (zeroth order) path and a spectral imaging (first order) path. The RAP then reflects photons in the spatial imaging path, and the custom-made DWP disperses the photons in the spectral imaging path. Finally, an electron-multiplying charge-coupled device (EMCCD) (iXon Ultra 897, Oxford Instruments) simultaneously detects all of the photons in the zeroth order and first order paths for further processing.

### 2.2 MC Simulation Process

Figure 1(b) shows our MC simulation process, from the generation of a blinking event by individual fluorophores to image detection. We start from the ground truth of N photons emitted in a single frame from one photoswitching event with parameters x, y, and λ, where N photons are either sampled from a lognormal function or assumed to be a constant value, x and y are the spatial positions of each photon, and λ is the wavelength of each photon sampled from the emission spectra. Because BSs often have wavelength-dependent splitting ratios and introduce photon losses, we pass the N photons through an efficiency function ε(λ) to determine whether each photon goes to the zeroth order path or first order path or is lost. Photons in the zeroth order path will be detected directly by the EMCCD, whereas photons in the first order path will pass through a dispersion function f(λ) to determine their wavelength-dependent shift along the x-axis ($x_{\mathrm{disp}}$), mimicking the behavior of the DWP. Next, we combine the locations of the photons, $X=x+x_{\mathrm{disp}}$ and Y=y, to determine the exact locations where each photon will be incident on the EMCCD array in Fig. 1(c). This signal is then discretized by individual EMCCD elements due to the finite pixel size (a) with varying intensities to give a signal (S) illustrated pictorially in Fig. 1(d). We further add a background signal consisting of Poisson-distributed background noises (B) and Gaussian-distributed readout noises^21^ (R), shown in Fig. 1(e). By summing the digitized signals from Figs. 1(d)–1(e), we apply signal amplification using a Gamma distribution, which is known to effectively model the behavior of an EMCCD^21^. This process yields Z, the simulated sSMLM frame shown in Fig. 1(f).

To obtain the subpixel peak location from the zeroth order spatial image, we fit the zeroth order image with a bivariate Gaussian function using the maximum likelihood estimation (MLE) method,^11^ giving us an estimate of the expected values of the peak locations x and y as x^ and y^, respectively. To obtain the spectral peak from the first order spectral image, we first calibrate the x-axis of the spectral graph with a spectral calibration procedure (detailed in Sec. 2.4), which allows us to calculate the wavelength values along the x-axis for each zeroth order localization. We then fit the spectrally calibrated image with either a univariate or bivariate Gaussian function to estimate the spectral peak (μ^λ) and spectral width (σ^λ) together with the spatial localization. The procedure is illustrated with a one-dimensional (1D) spectral graph in Fig. 1(g), where we integrated the signal intensity along the y-axis for clarity. An alternative method commonly used for spectral characterization is the spectral centroid method, which takes the weighted average of the different wavelengths detected to calculate a spectral centroid associated to each localization.^12^^,^^13^^,^^22^ In this study, we fitted the resulting image with a bivariate Gaussian function. This approach enhances noise rejection by taking advantage of the larger number of samples available in 2D data, as opposed to summing it along the y-axis in 1D. As the 2D Gaussian fit utilizes information from both dimensions, it provides a more accurate representation of the underlying distribution and consequently improves the robustness against noise.

To illustrate the functions N, λ, f(λ), and ε(λ) in Table 1 used in the simulation, we extracted the distribution of photon count and spectral signature of the commonly used SMLM dye Alexa Fluor 647 (AF647). Figure 1(h) shows the lognormal fitted distribution of photon counts N of AF647 extracted using the process described in Sec. 2.3.1. Figure 1(i) shows the spectral distribution λ of AF647 obtained with the procedure described in Sec. 2.3.2, and Fig. 1(j) shows a simulated point spread function (PSF) using a 2D-Gaussian function, which is a close approximation to the Airy disc.^23^ Figure 1(k) shows the spectral dispersion function, f(λ), experimentally measured using the procedure described in Sec. 2.3.3. Figure 1(l) shows the efficiency function, ε(λ), for BSs with reflectance (R):transmittance (T) of R30:T70 and R50:T50 experimentally measured using the procedure described in Sec. 2.3.4. Finally, we repeated the process 20,000 times to obtain the spatial and spectral precisions, defined as the standard deviation of x^ and μ^λ, respectively. We summarize the parameters used in the simulation in Table 1.

**Table 1:** Summary of simulation parameters.

Parameter	Meaning	Value/definition
*Vectors describing each photon in a single localization event*		
x, y	Vectors of calculated x and y positions	—
λ	Vector of wavelengths	—
θ	Vector of the paths taken by the photons after the BS	—
$x_{\mathrm{disp}}$	Vector of x dispersions due to the DWP module	—
*Matrices describing each pixel in the EMCCD pixel array*		
S	Signal on the pixel array	S; $S_{i,j}=\underset{i,j}{\sum }(x,y)$
B	Background noise on the pixel array	$B∼\mathrm{Po}(b_{\mathrm{bg}})$
R	Readout noise on the pixel array	$R∼N(0,b_{\mathrm{ro}})\times A/\mathrm{ADU}$
Z	Output on the EMCCD pixel array	Z=Γ(S+B,A)/ADU+R
$Z^{\prime }$	Output on the EMCCD pixel array after background subtraction	$Z^{\prime }=Z-A/\mathrm{ADU}\times b_{\mathrm{bg}}$
*Functions used in the simulation*		
N	PDF of the number of photons from fluorophore	$N∼\text{lognormal}(\mu ,\sigma^{2})$
λ	PDF of the wavelength of photon from the fluorophore spectrum	Custom function
ε(λ)	Efficiency function of the DWP module	ε(λ)∈0,1,−1
f(λ)	Spectral dispersion function of the DWP module	$f(\lambda )=x_{\mathrm{disp}}$
*Constants used in this paper*		
N	Number of photons from fluorophore	700
$b_{\mathrm{bg}}$	Mean number of background photons per pixel	15
$b_{\mathrm{ro}}$	Standard deviation of readout noise	$1.9\;e^{-}$
A	Gain on the EMCCD output	100
U	Analog to digital unit on the EMCCD	13.6
$a_{x}$, $a_{y}$	Pixel size	16 μm/px
$a_{\lambda }$	Spectral dispersion (linear equivalent)	4.3 nm/px
NA	Numerical aperture	1.49
$F_{\mathrm{EM}}$	Electron multiplying factor	$\sqrt{2}$
c	Conversion factor between FWHM and standard deviation	$2\sqrt{2\;\ln\;2}$.

### 2.3 Extraction of Parameters from Experimental Data

#### 2.3.1 Distribution of photon counts

To obtain physical values for the simulation, we extracted key parameters used in our MC simulation from our sSMLM experimental data. Figure 1(h) shows the histogram of the photon count N of AF647 molecules imaged at an exposure time of 10 ms. The photon counts were determined using ThunderSTORM,^24^ and the values obtained were fitted with a lognormal distribution.^25^

#### 2.3.2 Spectral distribution of fluorophores/microspheres

The example spectral signature of AF647 shown in Fig. 1(i) can usually be obtained from open-source databases, such as FPbase.^26^ For data unavailable in FPbase, such as for microspheres tested in this work, we perform a visible-infrared (Vis-IR) measurement of the emission spectra using the Nanodrop 3300 fluorospectrometer, which provides the bulk emission spectra of the microspheres.

#### 2.3.3 Spectral dispersion of the DWP module

We experimentally determined the spectral dispersion of the DWP module using a supercontinuum laser with an acousto-optic tunable filter (AOTF) to precisely adjust the output wavelength. We measured the angular deflection of the beam of the DWP module compared with the measurements without the DWP module inserted at different wavelengths. The experimental angular deflection curve was validated by the theoretical curve given by the Sellmeier equation,^27^ as shown in Fig. S1(a) in the Supplementary Material, with a good agreement. To better fit the theoretical curve, we used a rational function and found that the following equation works satisfactorily from 400 to 900 nm, given as

$$x_{\mathrm{disp}}=f(\lambda )=\frac{8495\xi +6132}{\xi^{2}+29.82\xi +74.03},$$ (1)

where $\xi =\frac{\lambda -650}{144.342}$ is a scaling factor. Because Eq. (1) is an invertible rational function, we find the inverse equation as

$$\lambda =f^{-1}(x_{\mathrm{disp}})=144.342(\frac{4247.5-14.91x_{\mathrm{disp}}-\sqrt{148.278x_{\mathrm{disp}}^{2}-120528x_{\mathrm{disp}}+18041300}}{x_{\mathrm{disp}}})+650$$ (2)

to obtain the wavelength from the angular deflection. Equation (2) adequately describes the scaling and translation optical transformations associated with using the DWP module. This is because, after any beam passes through the DWP module, the only transformations it can undergo are scaling (when the path length between the DWP module and the camera plane is changed) or translation (when the path length between the zeroth and first orders on the camera plane is changed). Following this, a linear equation between the linear dispersion and the photon wavelength can describe any spectral calibration curve of the DWP module as

$$x_{\text{scaled}}=af(\lambda )+b,$$ (3)

where a is the scaling factor and b is the translation factor. We use Eq. (3) to compute the spectral window of interest in the pixel for each localization detected in the spatial image. However, when we need to compute the spectral peak or spectral width, we combine the results of Eq. (2) and the inverse of Eq. (3) to get

$$\lambda =f^{-1}(\frac{x_{\text{scaled}}-b}{a}).$$ (4)

We compared the theoretical and the empirical curve in Fig. S1(b) in the Supplementary Material and found the angular error between the theoretical and the empirical curve to be <0.3%.

#### 2.3.4 Optical transmission efficiency of the DWP module

We evaluated the optical transmission efficiencies of the BS, RAP, and DWP by measuring the transmitted and reflected optical power ratios using a supercontinuum laser with an AOTF setup, as described in Sec. 2.3.3. We then calculated the efficiencies at different wavelengths by calculating the ratios of transmitted ($P_{T}$) and reflected ($P_{R}$) optical power to the total incident power ($P_{I}$). Because our AOTF adjustable spectral range was from 450 to 690 nm, we used the data to validate the Thorlabs datasheet for the BSs. To extend the spectral range beyond 690 nm for the RAP, we assumed that the efficiencies remain relatively constant. We utilized the average efficiency value measured for the range of 450 to 690 nm and applied it throughout our simulated spectral range of 450 to 800 nm. For the DWP, we extrapolated our results for efficiencies beyond 690 nm. In Fig. S2 in the Supplementary Material, we present the experimentally determined plots as dots for each optical component, and the corresponding dotted lines represent the simulated curves.

### 2.4 Post-Processing of EMCCD Images

#### 2.4.1 Spectral calibration

To calibrate the spectral dispersion of the DWP, we used a custom-made nanohole array as a static target for spectral calibration and five bandpass filters (Table S1 in the Supplementary Material). The nanohole array (Fig. S3 in the Supplementary Material) was fabricated on a gold-plated glass coverslip using a Helios Nanolab 600 Focused ion beam-scanning electron microscope. To obtain the average spectral dispersion ($x_{\mathrm{disp}}$) of each hole at different wavelengths, we imaged the nanohole array through the 5 bandpass filters, which have center wavelengths of 532, 605, 635, 685, and 750 nm. Based on these measurements, we performed a linear fit using Eq. (4) to determine the spectral deflection at different wavelengths of the DWP module.

#### 2.4.2 Correcting for nonlinearity in spectral fits in the DWP module

**Fig. 2:**
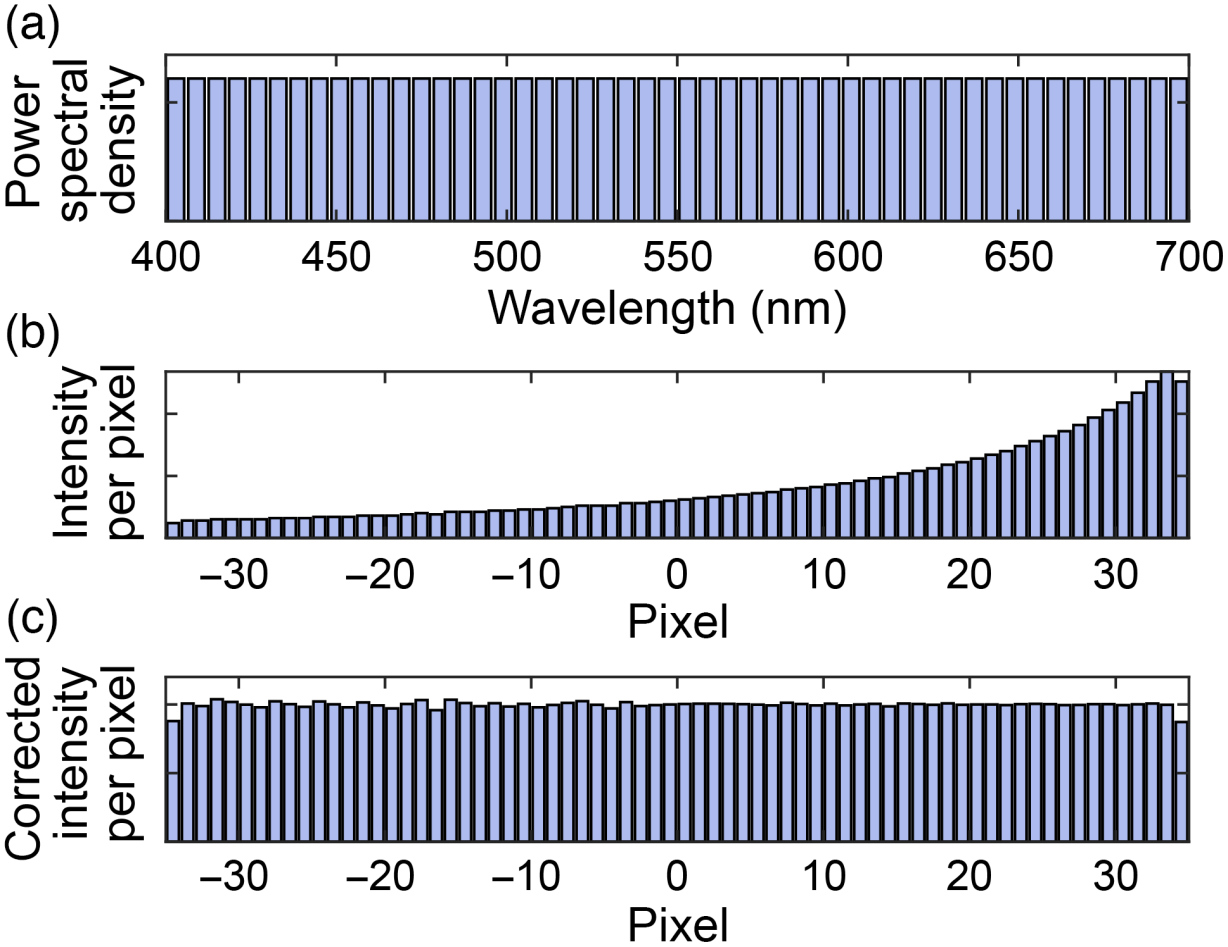
Illustration of the correction process of nonlinearity in the spectral dispersion with DWP. (a) Illustration of the wavelength distribution of an ideal uniformly distributed spectral density light source; (b) power density detected by the EMCCD after the DWP; and (c) corrected power density.

To accurately fit the spectra obtained from the DWP module, it is crucial to consider the inherent nonlinearity in spectral dispersion. As shown in Fig. 2(a), a hypothetical light source with a uniform spectral power density undergoes spectral dispersion after passing through the DWP module, resulting in non-uniform intensity across the image sensor, as shown in Fig. 2(b). This effect is particularly noticeable in regions with lower spectral dispersion, where the incident power is greater. Consequently, fitting a Gaussian curve to the spectrum would result in a bias toward the longer wavelengths due to the higher intensity in the region. To account for this spectral bias, we normalized the spectra with the spectral dispersion, as shown in Fig. 2(c), which results in a corrected image with uniform intensity throughout the image sensor. This normalization process corrected the nonlinear spectral dispersion in the DWP module and ensured accurate spectral fitting.

### 2.5 Validation with Fluorescence Microspheres

To validate our MC model, we imaged microspheres with emission peaks at 560, 580, 605, 645, and 720 nm (F8800, F8794, F8801, F8806, and T8870, Invitrogen) deposited on #1.5-thick glass coverslips (12-541-B, Fisher Scientific). The other physical properties of these microspheres are detailed in Table S2 in the Supplementary Material. We first washed the glass coverslips with phosphate-buffered saline (PBS) and coated the coverslips with a 0.001% poly-L-lysine solution for 5 min. We then washed the coverslips with PBS and stained the glass coverslips with a diluted sample of each microsphere for 1 h. Next, we washed off the unbounded microspheres with PBS three times and added a single drop of antifade buffer (P36961, Invitrogen). Finally, we sealed the coverslips with black nail polish (114-8, Ted Pella) and left them overnight to dry.

We first imaged the microspheres without the DWP module to obtain the photon count of each nanosphere and then imaged the same microspheres with the DWP module for 100 frames. To obtain the spatial localization precision, we processed the 100 frames of microspheres using ThunderSTORM^24^ and treated the standard deviation from x^ calculated from the 100 frames as the localization precision. To obtain the spectral precision, we identified a region of interest from 450 to 800 nm relative to the zeroth order image after spectral calibration using the method previously described.^5^^,^^13^^,^^22^ This region of interest gives us a spectral profile similar to the one seen in Fig. 1(f), which we use to analyze the spectral signature of the fluorophore further. Next, we subtracted the background using averaged neighboring windows of the first order image. Then, we performed a least-squares fit of the spectral profile with a bivariate Gaussian function to identify the spectrum corresponding to the zeroth order blinking.

## 3 Results and Discussion

### 3.1 Validating MC Simulation with Theoretical Analyses

To validate our MC model, we first benchmarked the precisions generated from our model against theoretical models of spatial localization precision Δx defined as^11^

$$\Delta x^{2}=\frac{\sigma_{x}^{2}+\frac{a_{x}^{2}}{12}}{N}(1+4\tau +\sqrt{\frac{2\tau }{1+4\tau }}),$$ (5)

where $\sigma_{x}$ is the sigma of the PSF spot along x, $a_{x}$ is the pixel size, and N is the photon count. τ is the background correction factor and is defined as $\tau =\frac{2\pi (b_{\mathrm{bg}}+b_{\mathrm{ro}}^{2})(\sigma_{x}^{2}+\frac{a_{x}^{2}}{12})}{Na_{x}^{2}}$, where $b_{\mathrm{bg}}$ is the mean number of background photons per pixel, $b_{\mathrm{ro}}$ is the standard deviation of readout noise, and the other parameters are as defined previously. The spectral precision $\Delta \mu_{\lambda }$ is defined as^12^

$$\Delta \mu_{\lambda }^{2}=F_{\mathrm{EM}}^{2}(\frac{\sigma_{\lambda }^{2}}{c^{2}N}+\frac{1024b_{\mathrm{bg}}\sigma_{\lambda }^{3}\sigma_{y}}{3a_{y}a_{\lambda }c^{3}N^{2}})+\frac{1024b_{\mathrm{ro}}^{2}\sigma_{\lambda }^{3}\sigma_{y}}{3a_{y}a_{\lambda }c^{3}N^{2}}+\frac{a_{\lambda }^{2}}{12},$$ (6)

where $F_{\mathrm{EM}}$ is the correction factor of the EMCCD equal to $\sqrt{2}$, $\sigma_{\lambda }$ is the full-width-at-half-maximum (FWHM) of the spectral bandwidth, c is the conversion factor between FWHM and the standard deviation of a Gaussian function equal to $2\sqrt{2\;\ln\;2}$, $a_{\lambda }$ is the spectral dispersion, and all other parameters are as defined above.

Equation (5) describes the performance limit of the MLE when fitting the PSF into an approximate 2D Gaussian curve, also referred to as the Cramér–Rao lower bound.^11^ Equation (6) assumes (1) a uniform spectral dispersion, (2) a Gaussian spectral shape with a spectral peak of $\mu_{\lambda }$ and a spectral FWHM of $\sigma_{\lambda }$, and (3) spectral centroid in the calculation of the spectral precision.^12^ The spectral centroid method takes the weighted average of the intensities captured in the pixels associated with different wavelengths, yielding a weighted representation of the spectra recorded.

To provide a fair comparison to validate our simulation, we matched the simulation based on the assumptions of Eq. (6) listed above. We computed the spatial localization precision, defined as the standard deviation of the localized spot from the true location, and the spectral precision, defined as the standard deviation of the spectral centroid or the fitted spectral peak. We performed MC simulations under the following conditions: N=700, $b_{\mathrm{bg}}=15$, spectral peak=680  nm, and spectral FWHM=41  nm, using a linear spectral dispersion of 5 nm/px, unless otherwise stated.

**Fig. 3:**
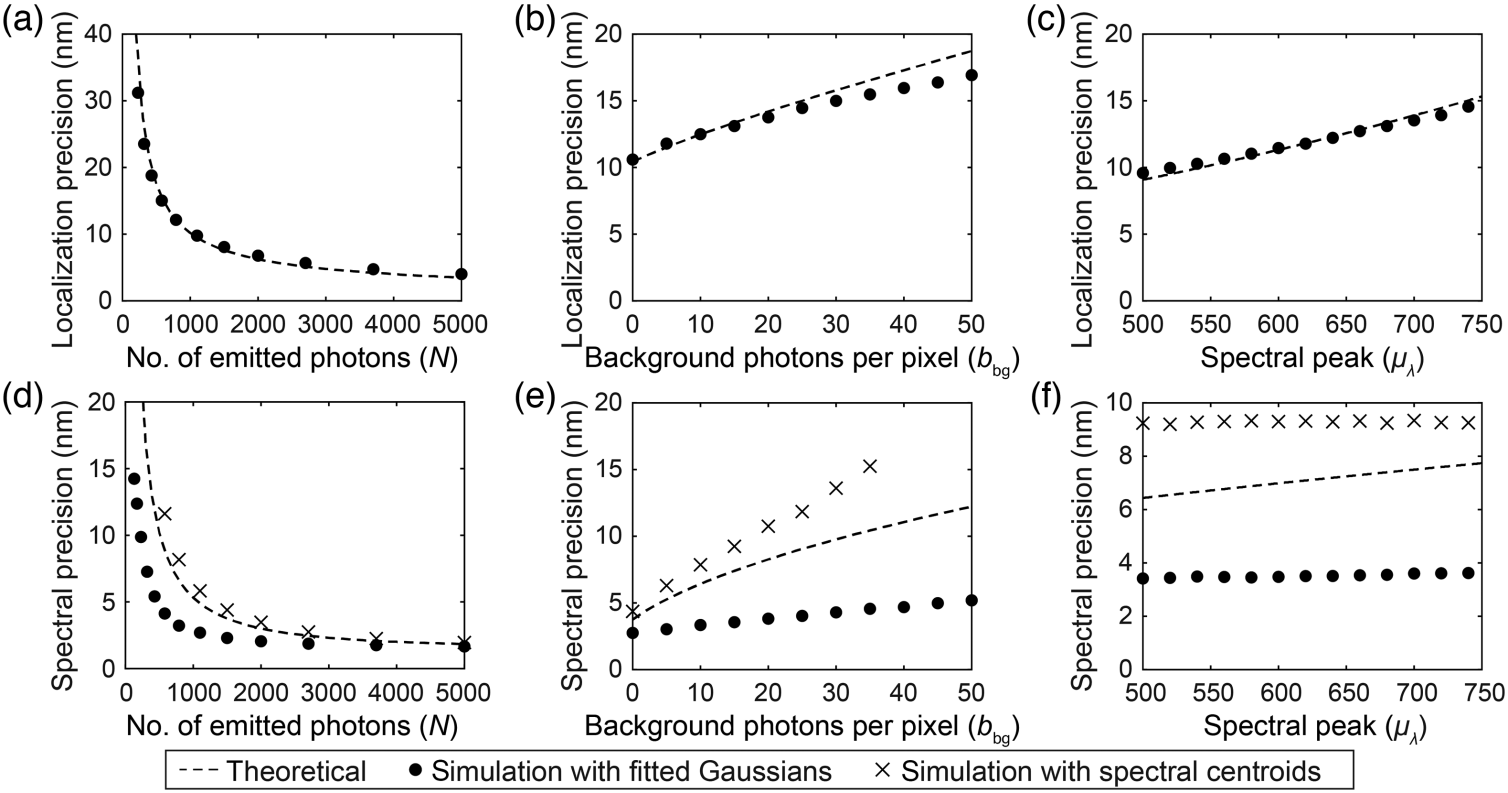
Comparison of localization precisions between theoretically predicted results (dashed line) and simulation results (dots) with respect to (a) photon count, (b) background photons, and (c) emission spectral peak. Comparison of spectral precisions between theoretically predicted results (dashed lines), simulated results calculated with spectral centroids (crosses), and simulated results calculated with a bivariate Gaussian fit (dots) with respect to (d) photon count, (e) background photons, and (f) spectral peak.

Figure 3 shows the influence of photon counts N, background photons $b_{\mathrm{bg}}$, and emission spectral peak $\mu_{\lambda }$ on lateral localization and spectral precisions. In Figs. 3(a)–3(c), we compared the theoretical localization precisions calculated with Eq. (5) with our MC simulation, with mean absolute errors of 2.5, 0.8, and 0.3 nm relative to the theoretical values, respectively. Similarly, in Figs. 3(d)–3(g), we compared the theoretical spectral precisions calculated with Eq. (6) with our MC simulation using the spectral centroid method, with mean absolute errors of 2.1, 7.6, and 2.2 nm, respectively. For the spectral fitting method, the simulated precisions are consistently better, with mean absolute errors of 6.3, 4.6, and 3.6 nm for Figs. 3(d)–3(g), respectively.

In Figs. 3(a) and 3(d), we observe that increasing the number of emitted photons from 1500 to 5000 improved the localization precision from 8.1 to 4.0 nm. Similarly, the spectral precision improved from 4.4 to 2.0 nm with the spectral centroid method and 3.2 to 1.6 nm with the spectral fitting method. With a limited photon budget, splitting the photon budget between zeroth order and first order images can affect the tradeoff between the localization precision and spectral precision.

In Figs. 3(b) and 3(e), increasing background photons from 0 to 30 per pixel worsens the localization precision from 10.6 to 15.0 nm. Similarly, spectral precision worsens from 4.4 to 13.6 nm with the spectral centroid method and from 2.7 to 4.3 nm with spectral fitting. The spectral fitting method is more robust against noise because it estimates the background term by fitting for and averaging over all pixels available in the spectral window, reducing the variation in the estimated spectral peak μ^λ. By contrast, the spectral centroid method takes the weighted average of the spectral window, which is more susceptible to noise variations. In addition, accurately estimating the background level can be challenging,^28^ which can result in biases to the spectral centroid value. A positive bias can result from the presence of background contribution to the spectral window, which can shift the spectral centroid value toward the mean of the spectral window and reduce the spectral precision. Hence, we prefer the bivariate Gaussian fitting method due to its robustness in estimating the spectral peak μ^λ and calculating for spectral precisions.

In Figs. 3(c) and 3(f), we observe that, as the center wavelength of the emission peak increases from 500 to 740 nm, the localization precision deteriorates from 9.6 to 15.0 nm. Simultaneously, the spectral precision is stable at ∼9.2  nm with the spectral centroid method and at 3.5 nm with the spectral fitting method. This effect is more pronounced in Fig. 3(c), where the wider FWHM of the PSF results in improved localization precision. However, in Fig. 3(f), a spectral emission FWHM of 47 nm plays a more significant role in determining the overall width of the first order image with added spectral dispersion. Overall, our MC model agrees well with the theoretical predictions, with a worse-case average absolute error of 2.5 nm for lateral precision and 2.1 nm for the spectral precision with the spectral centroid method across all cases.

### 3.2 MC Simulation of Different BSs

**Fig. 4:**
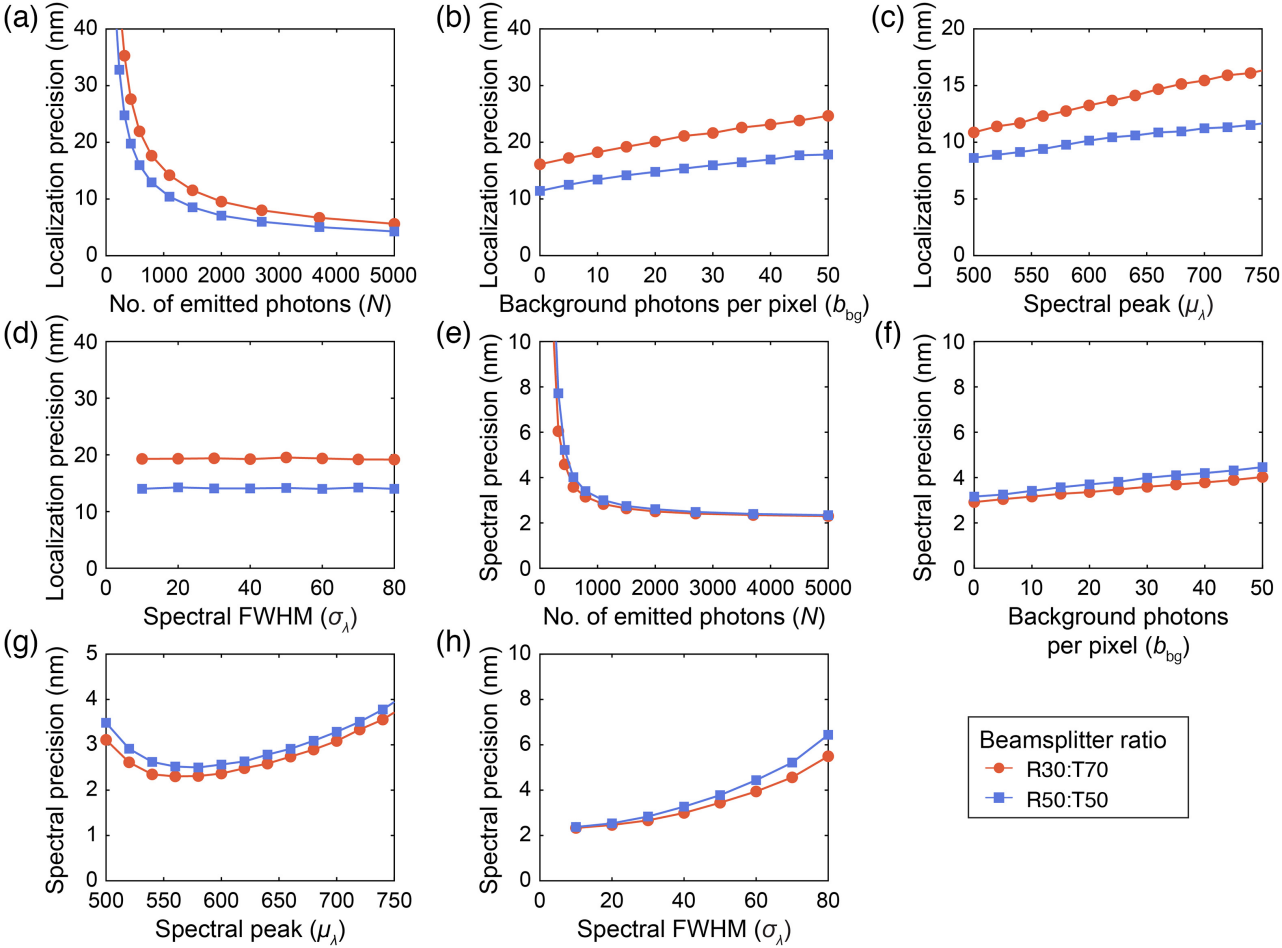
Comparison of localization precisions between an R50:T50 (orange dots) and R30:T70 (blue squares) BS with respect to (a) photons count, (b) background photons, (c) spectral peak, and (d) spectral FWHM. Comparison of spectral precisions between an R50:T50 (orange dots) and R30:T70 (blue squares) BS with respect to (e) photons count, (f) background photons, (g) spectral peak, and (h) spectral FWHM.

After validation, we used the MC model to predict the performance of different BSs used in the DWP module. Figure 4 shows how photon counts N, background photons $b_{\mathrm{bg}}$, and emission spectral peak $\mu_{\lambda }$ affect spatial localization and spectral precisions with different BSs. We conducted MC simulations under the following conditions: N=700, $b_{\mathrm{bg}}=15$, $\mu_{\lambda }=680\;\mathrm{nm}$, and emission spectral FWHM=41  nm, and we used the DWP system with different BS ratios unless otherwise stated.

Figures 4(a)–4(d) show that using an R30:T70 BS results in a 35% worse localization precision on average compared with an R50:T50 BS. However, Figs. 4(e)–4(h) demonstrate that an R30:T70 BS provides 8% better spectral precision on average compared with an R50:T50 BS. This is because the R30:T70 BS redirects more of the photon budget toward the spectral channel, which leads to lower spectral precisions, as shown in Fig. 3(d). The tradeoff between spectral and spatial localization precisions is especially prominent in photon-limited photoswitching events when N<1000, as shown in Figs. 4(a) and 4(e), where we observed up to a 12.3 nm worsening in the localization precision and up to 2.7 nm improvement in the spectral precision for N=320. From Fig. 1(h), many photoswitching events of AF647 have N<1000, which suggests that the change of BS can play a significant role in optimizing the accuracy in the classification of different fluorophores.

Increasing the number of background photons from 0 to 50 per pixel leads to an increase in the localization and a minimal increase in the spectral precision in both BSs. In Fig. 4(b), the localization precision worsens from 11.4 to 17.8 nm for the R50:T50 BS and from 16.1 to 24.7 nm for the R30:T70 BS. In Fig. 4(f), the spectral precision worsens slightly from 3.2 to 4.5 nm for the R50:T50 BS and from 2.9 to 4.0 nm for the R30:T70 BS. Overall, switching from an R50:T50 BS to an R30:T70 BS led to an average worsening in the localization precision by 5.6 nm and an improvement in the spectral precision by 0.3 nm when the number of background photons per pixel is varied.

Figure 4(c) shows that, as the spectral peak changes from 500 to 750 nm, the localization precision worsens from 8.6 to 11.8 nm for the R50:T50 BS and from 10.9 to 16.6 nm for the R30:T70 BS. This worsening is attributed to the change in PSF’s FWHM, as observed in Fig. 3(c). However, Fig. 4(g) reveals a nonlinear relationship between the spectral precision and spectral peak, unlike in Fig. 3(f), with mean values of 3.0 nm for the R50:T50 BS and 2.8 nm for the R30:T70 BS. This nonlinearity results from the nonlinear spectral dispersion of the DWP system, as shown in Fig. 1(k), causing spectral precision to vary based on the emission wavelength. Overall, switching from an R50:T50 BS to an R30:T70 BS led to an average worsening in the localization precision of 3.5 nm, an average improvement in the spectral precision of 0.2 nm, and up to a 0.4 nm improvement in the spectral precision at a spectral peak of ∼540  nm.

Figure 4(d) shows that the localization precision remains relatively stable when the FWHM spectral bandwidth increases from 10 to 80 nm. Figure 4(h) shows that the spectral precision worsens from 2.3 to 6.4 nm for the R50:T50 BS and from 2.3 to 5.5 nm for the R30:T70 BS. This increase is due to the broader spread of the first order image on the EMCCD, with results in a lower signal-to-noise ratio in the detected spectra for a fixed photon count N, and, consequently, poorer precision. However, the localization precision remains mostly unaffected as the PSF does not change significantly compared with the spectral shape. Overall, switching from an R50:T50 BS to an R30:T70 BS led to an average worsening in the localization precision by 5.2 nm and an average improvement of the spectral precision by 0.4 nm.

### 3.3 Testing MC Simulation Against Experimental Data

**Fig. 5:**
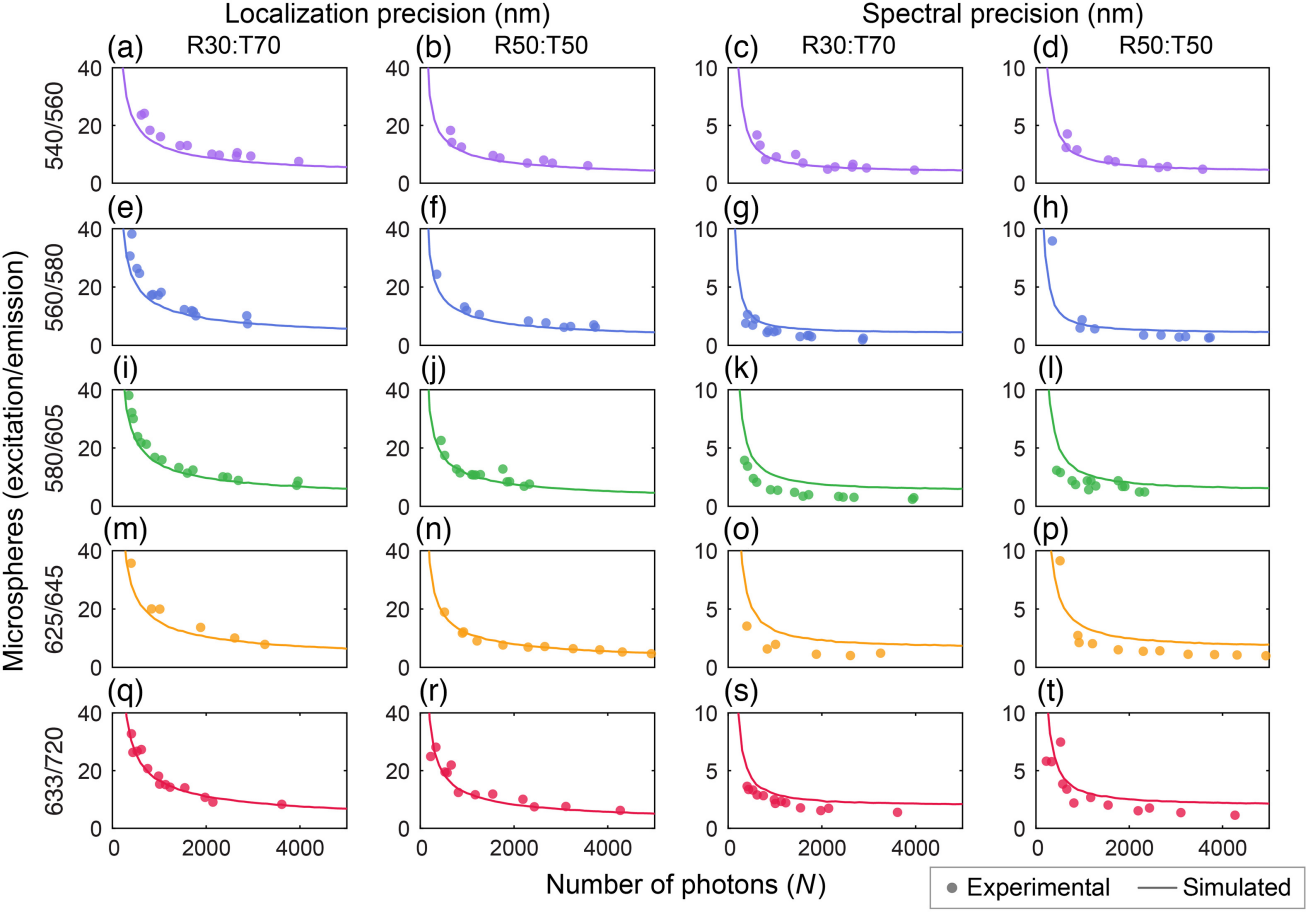
Localization and spectral precisions of different microspheres under varying numbers of emitted photons with DWP-based sSMLM system: (a)–(r) localization precisions and (c)–(t) spectral precisions. Each row shows the localization and spectral precisions of a single species of microspheres. The BS used in each case is indicated at the top of each column.

Figure 5 tests our MC simulation against experimental results obtained by imaging microspheres. The results demonstrate a good match between the MC-DWP simulation and our experimental data for the localization precision, as shown in Figs. 5(a)–5(r), with an average absolute error of 2.7 nm for the R30:T70 BS and 1.5 nm for the R50:T50 BS. However, for the spectral precision shown in Figs. 5(c)–5(t), we observed a slightly higher error, with an average of 0.9 nm for the R30:T70 BS and 1.0 nm for the R50:T50 BS, compared with the simulation.

The difference in the spectral precision can be attributed to two factors: (1) spectral variations within individual microspheres and (2) imperfect background estimations in the experimental results. Although the simulation results used the bulk spectrum of the microspheres to generate the data, the experimental results measured the spectra of individual microspheres. At the molecular level, individual spectra are not readily available, and it is known that, when adding two unequal Gaussian probability density functions (PDFs), the result is a PDF with a broader width. Because the individual spectral signatures of the microspheres differ from the bulk phase due to heterogeneity,^22^ their narrower widths likely collectively combine to produce a broader bulk phase spectral signature. As shown in Fig. 4(h), increasing the spectral FWHM by 10 nm results in ∼10% degradation in the spectral precision. The influence of spectral heterogeneity present in our experimental findings explains the slightly improved calculated spectral precision in the experiments compared with the simulation results.

Furthermore, background estimation and subtraction can be challenging and biased because the microspheres are not photoswitching. During data processing, background subtraction may have caused some microspheres to have consistently lower spectral precisions than the simulated values, resulting in systematic errors in the calculated spectral precision.^28^ Nonetheless, our MC-DWP simulation provides a reasonable estimate of the performance of the different nanospheres.

Finally, our analysis does not consider changing the spectral dispersion of the system because the DWP module was designed using an optimal spectral dispersion range of 4 to 6, which minimizes the spectral uncertainty when N ranges from 1000 to 2000.^12^ This N range is the photon budget available for most fluorophores commonly used in biological imaging applications. Therefore, the beamsplitting ratio and fluorophore combinations are key to maximizing system performance. In particular applications, such as the spectroscopic analysis of dyes,^22^ in which the photon budget is fixed, we may omit the BS and use the entire available photon budget for spectroscopic analysis. In this case, the only way to tune the spectral performance of the system is to change the spectral dispersion.

## 4 Conclusion

We developed a physically informed MC model for predicting the imaging performance of DWP-based sSMLM systems. Our model accurately predicts both localization and spectral precisions, as well as spectral peaks and widths in fluorescent microspheres, which provides a theoretical foundation for optimizing the performance of multiplexed imaging. By simulating the DWP-based system using different BSs, we found that an R30:T70 BS can reduce the spectral precision by up to 14%, albeit with a penalty of 35% in the localization precision on average. Our work can guide the optimization of imaging parameters for common fluorophore combinations to maximize both localization and spectral performance. Moreover, our model can generate ground-truth data for different fluorophores, which could improve machine-learning algorithms for more accurate fluorophore identification.^5^^,^^29^ Although there are limitations in the agreement between our model and experimental data due to the spectral heterogeneity of individual microspheres, we are exploring new theoretical and simulation models that can potentially describe the spectral heterogeneity of microspheres and fluorophores, which could further improve imaging performance and accuracy.

## Code, Data, and Materials Availability

The source code for the Monte Carlo simulation can be found at https://github.com/FOIL-NU/MC-DWP.
