# Supplementary material for: Physically informed Monte Carlo simulation of dual-wedge prism-based spectroscopic single-molecule localization microscopy
Source: J Biomed Opt. 2023 Oct 3;29(Suppl 1):S11502. doi: 10.1117/1.JBO.29.S1.S11502 (PMC10546470; doi:10.1117/1.JBO.29.S1.S11502)
Supplement: Supplementary file 1 [file JBO_029_S11502_SD001.pdf]

Physically informed Monte Carlo simulation of dual-wedge prism-based spectroscopic single-molecule localization microscopy

Wei-Hong Yeo,[a] **Cheng Sun,**[b] **Hao F. Zhang**[a,*]
[a] Northwestern University, Department of Biomedical Engineering, Evanston, United States
[b] Northwestern University, Department of Mechanical Engineering, Evanston, United States

*Hao F. Zhang, E-mail: hfzhang@northwestern.edu

Appendix A: Supplemental Material

Table S1 Specifications and source of bandpass filters used for spectral calibration.

Center wavelength (nm)	Full width at half maximum (nm)	Product Code	Manufacturer
532	1	FL532-1	Thorlabs
605	15	FF01-605/15-25	Semrock
635	10	FL635-10	Thorlabs
685	10	86-738	Edmund Optics
750	10	FB750-10	Thorlabs

Table S2 Optical and physical properties of Invitrogen microspheres used in the experiment.

Excitation wavelength (nm)	Emission wavelength (nm)	Diameter of microspheres (μm)	Product Code
540	560	0.1	F8800
565	580	0.04	F8794
580	605	0.1	F8801
625	645	0.2	F8806
633	720	0.04	T8870

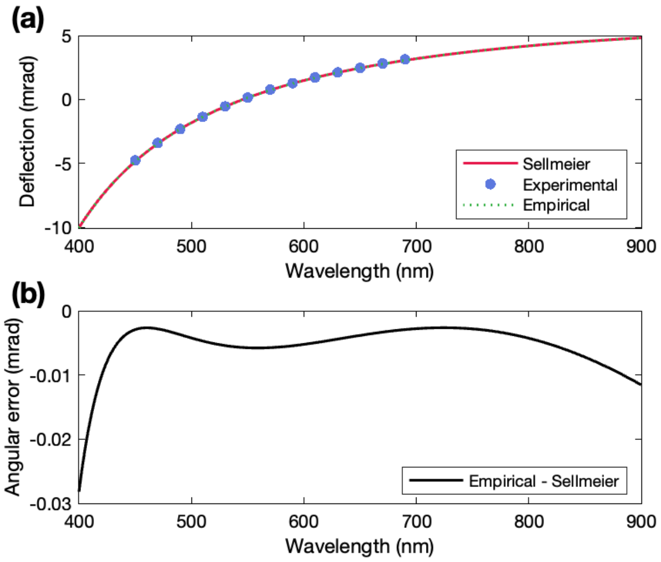

Fig. S1 (a) Plot of the deflection of the DWP module determined experimentally (blue dots); theoretically using the Sellmeier equation (red solid line); and empirically using the rational best fit line (green dotted line). (b) Plot of the angular error between the empirical rational best fit line and the theoretical curve derived from Sellmeier equation.

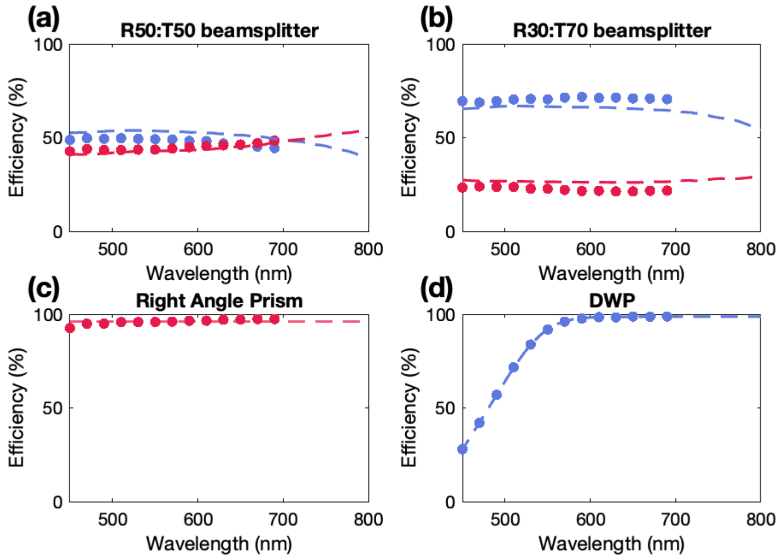

Fig. S2 Plots of efficiency curves of (a) R50:T50 beamsplitter, (b) R30:T70 beamsplitter, (c) right-angle prism, (d) dual wedge prism. We obtained efficiency data using a supercontinuum laser with an AOTF setup and shown our data as dots in the graph. The dotted lines for (a)-(b) refer to the Thorlabs datasheet; for (c), the average over the experimental datapoints acquired; and for (d), the extrapolated curve from the experimental datapoints acquired. In all graphs, blue datapoints refer to transmission efficiencies, and red datapoints refer to reflection efficiencies.

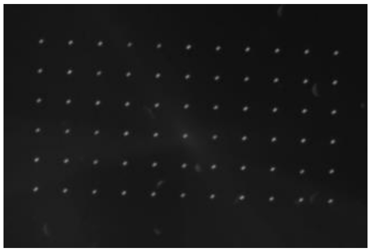

Fig. S3 A sample image of our nanohole array.
